# Extracts of *Knoxia roxburghii* (Spreng.) M. A. Rau Induce Apoptosis in Human MCF-7 Breast Cancer Cells via Mitochondrial Pathways

**DOI:** 10.3390/molecules27196435

**Published:** 2022-09-29

**Authors:** Xiao-Jiao Chen, Xin-Ying Pu, Xue-Mei Pu, Xue Li, Zhi-Bo Liu, Mi-Jia Mei, Xin-Ge Wang, Fan Zhang, Bin Qiu, Jie Yu

**Affiliations:** Yunnan Key Laboratory of Southern Medicine Utilization, College of Pharmaceutical Science, Yunnan University of Chinese Medicine, 1076 Yuhua Road, Chenggong District, Kunming 650500, China

**Keywords:** *Knoxia roxburghii* (Spreng.) M. A. Rau, phytochemicals, breast cancer, apoptosis, extract, UHPLC-LC/MS

## Abstract

*Knoxia roxburghii* (Spreng.) M. A. Rau (KR) is a plant clinically used in traditional Chinese medicine (TCM) for the treatment of cancer. The study objectives were to examine the effects of KR extracts, petroleum ether (PET), ethyl acetate (EtoAc), butanol (n-BuOH), and H_2_O-soluble fractions (HSF) of the 75% EtOH extraction on A549 (non-small cell lung cancer), HepG2 (liver cancer), HeLa (cervical cancer), MCF-7 (breast cancer), and L02 (normal hepatocyte) cells. It was found that HSF exhibited the strongest cytotoxic activity against MCF-7 cells, and was accompanied by reduced mitochondrial transmembrane potential, increased levels of intra-cellular reactive oxygen species (ROS) and activated caspases, and upregulated pro-apoptotic and downregulated anti-apoptotic proteins. LC-MS analysis further showed that HSF primarily consisted of calycosin, aloe emodin, rein, maackiain, asperuloside, orientin, vicenin-2, and kaempferide, which have been mostly reported for anti-tumor activity in previous studies. In summary, the current study illustrated the effect, mechanism, and the potential major active components of KR against breast cancer.

## 1. Introduction

More than half of all known anti-cancer drugs are derived from herbs [1]. Natural products are still one of the best sources for the discovery of new drugs [2]. Since its discovery in 1970 in the Pacific yew tree, *Taxus brevifolia*, taxol is one of the most clinically valuable highly oxygenated terpenoids [3]. In addition to the anti-cancer compounds vinblastine and vincristine, the plant Catharanthus roseus contains several valuable terpenoid indole alkaloids (TIAs) [4]. Compounds from plants are usually less toxic to normal cells than their synthetic counterparts and have alternative mechanisms to promote cancer cell death. Moreover, it is reported that the photochemical mixtures found in various balanced diets play a synergistic role, leading to enhanced anti-cancer biological activity and health benefits, which cannot be achieved with the intake of single active compounds [5,6]. The availability of bioactive anti-cancer compounds in plants has sparked an unprecedented interest in their study by researchers [7].

*Knoxia roxburghii* (Spreng.) M. A. Rau (KR) belongs to the Rubiaceae family. In China, it is known as ‘Zi Daji’. In recent years, clinical research on TCM has found curative effects of KR on cancers, carbuncles, diarrhea, and ascites, as well as chronic pharyngitis and schizophrenia [8,9,10]. It is also used as one of the major components in many Chinese herbal formulas, such as the Zijin tablet, which have been proven by modern pharmacology to have anti-tumor effects [11]. 

Cytotoxicity experiments showed that the main anthraquinones in KR exhibited a certain level of cytotoxicity to tumor cells [12]. In recent years, it has also been employed for the inhibition of *Staphylococcus aureus*, *Mycobacterium tuberculosis*, and *Pseudomonas aeruginosa* [13]. It has been reported that KR inhibited the formation of advanced glycation end products and rat lens aldose reductase in vitro [14]. However, no in vivo or in vitro research has been conducted to compare its effects on various types of cancers. 

KR contains a variety of medicinal chemical components, mainly anthraquinones and non-anthraquinones, such as triterpenes, lignans, and phenolic acids. Anthraquinones are also important sources of anti-tumor drugs [15]. TCM rich in anthraquinone, such as *Rheum officinale*, *Polygonum cuspidatum*, and *Radix rubiae*, can inhibit the metastasis of tumor cells. For example, emodin is an anthraquinone component of *Rheum officinale* that has been proven to inhibit the occurrence and development of various tumors [16,17]. Triterpenoids are the most abundant natural products found in nature. Many studies have shown that triterpenoids have obvious inhibitory effects on breast cancer, lung cancer, rectal cancer, and cancers of the central nervous system [18,19,20]. Ursolic acid, damnacanthal, bergapten, and 2α,3α,24-trihydroxy-12-alkene-28-ursolic acid have been isolated from KR [21,22], and have been proven to have anti-tumor effects [14,23,24,25,26,27,28].

Nonetheless, other types of cancer cells are not known to be susceptible to extract-mediated cytotoxicity. A detailed investigation of specific constituents of KR extracts and their mode of action has not been provided in any of these studies. Thus, molecular mechanisms underlying this activity are unknown at present. This study was conducted to assess the anti-cancer effects of KR extracts in vitro. Organic-soluble fractions containing potentially bioactive compounds were prepared by sequential extraction using organic solvents with increasing polarity. Then, the CCK-8 kit was used to determine the KR fractions’ inhibitory activity against different cancer cells (A549, HepG2, HeLa, and MCF-7 cells) and L02 cells. Finally, based on the experimental results, MCF-7 cells and H_2_O-soluble fractions (HSF) exhibiting the best inhibitory effects were screened out, and their chemical composition and mechanism were studied. HSF was examined in this study for its effect on proliferation, apoptosis, mitochondrial membrane potential (MMP) collapse, and reactive oxygen species (ROS) formation in breast cancer cells, and it has been hypothesized that a mitochondrial pathway might be required to mediate its anti-breast cancer effect.

## 2. Results

### 2.1. Effect of the Different Fractions on the Proliferation of Cancerous Cells 

**Figure 1:**
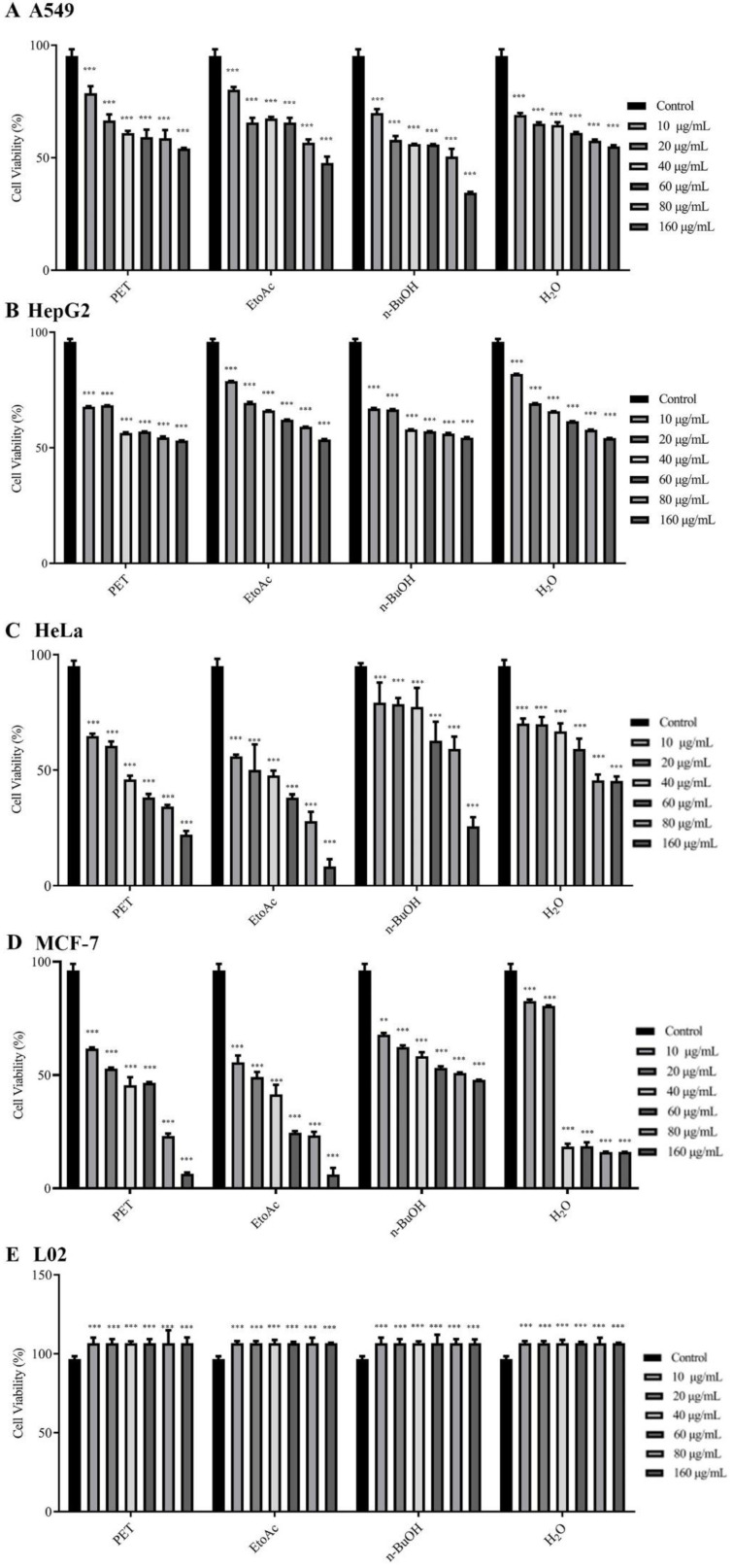
Effects of different fractions (PET, EtoAc, n-BuOH, and H_2_O) on A549, HepG2, HeLa, MCF-7, and L02 cell viability. (**A**) Effects of different fractions on A549 cell viability. (**B**) Effects of different fractions on HepG2 cell viability. (**C**) Effects of different fractions on HeLa cell viability. (**D**) Effects of different fractions on MCF-7 cell viability. (**E**) Effects of different fractions on L02 cell viability. ** *p* < 0.01, *** *p* < 0.001 vs. control group.

Cell growth was inhibited in a dose-dependent manner by all fractions in A549, HepG2, HeLa, and MCF-7 cells (Figure 1). Notably, as measured by the IC_50_ value of 28.84 ± 0.60 μg/mL (Table 1), HSF had the most potent cytotoxic effect against MCF-7 cells. In contrast, the L02 cells were not affected by any of the treatments. According to our hypothesis, it was selectively cytotoxic in various cancer cell lines but was not toxic to normal cells.

Consequently, MCF-7 was chosen for subsequent mechanistic studies, since it is the most sensitive to HSF cytotoxicity.

### 2.2. Identification of Compounds Present in HSF of KR 

A total of eight compounds, including flavonoids, anthraquinones, and triterpenoids, were identified from the HSF of KR extracts via LC-MS analysis (Table 2). Among these, calycosin, aloe emodin, rhein, and maackiain reportedly exhibit effects against breast cancer [29,30,31], while asperuloside, orientin, vicenin-2, and kaempferitrin were proven to exhibit anti-tumor activity [32,33,34,35,36]. Additionally, 3-hydroxymorindone and rubiadin were found to occur in HSF through a comparison with reference compounds. At present, asperuloside has been reported to be isolated and identified from KR [37]. The remaining compounds are unknown. 

### 2.3. Effect of HSF on Apoptosis Staining of MCF-7 Cells

**Figure 2:**
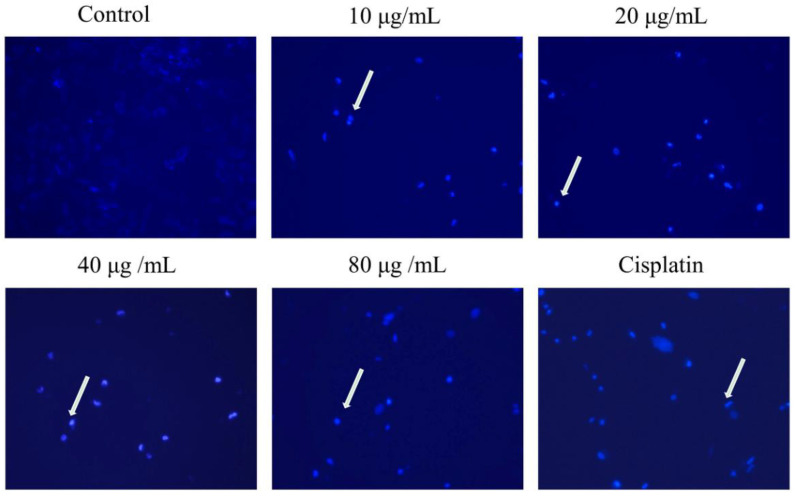
MCF-7 cell apoptosis staining. Hoechst 33258 staining revealed changes in nuclear morphology. The phase contrast inverted microscopic images of the experimental groups (magnification, ×10). Apoptotic nuclei as indicated by the white arrow.

Experimental and control groups differed in fluorescent microscopic analysis, with control nuclei being blue. As the concentration of HSF increased, the nuclei of MCF-7 cells underwent typical morphological changes such as nucleus condensing and fragmentation (Figure 2). 

### 2.4. Effect of HSF on the Apoptosis Rate of MCF-7 Cells

**Figure 3:**
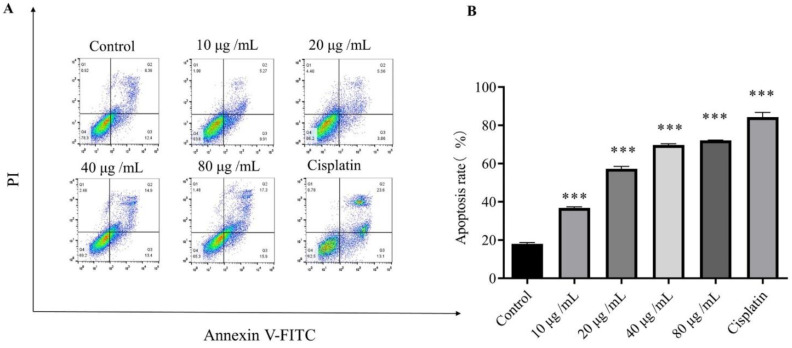
Effect of HSF on apoptosis of MCF-7 cells. Annexin V-FITC and PI staining were detected by flow cytometry. (**A**) Flow cytometry for the analysis of cell apoptosis. (**B**) Apoptosis rate. *** *p* < 0.001 vs. control group.

After treatment with HSF, MCF-7 cells were stained with Annexin V-FITC/PI to determine the mode of cell death. Dot plot analyses are displayed in the figure below (Figure 3A). In comparison with the control group, the percentage of cells in early (Annexin-V positive and PI negative) and late apoptosis (Annexin-V positive and PI positive) was significantly increased, and the number of living cells (Annexin-V negative and PI negative) was significantly reduced after HSF treatment, as compared to the control. Collectively, it was found that the percentage of early and late apoptotic cells increased with dose (Figure 3B). It was found that HSF effectively induced apoptosis in MCF-7 cells.

### 2.5. Effect of HSF on MMP Levels in MCF-7 Cells

**Figure 4:**
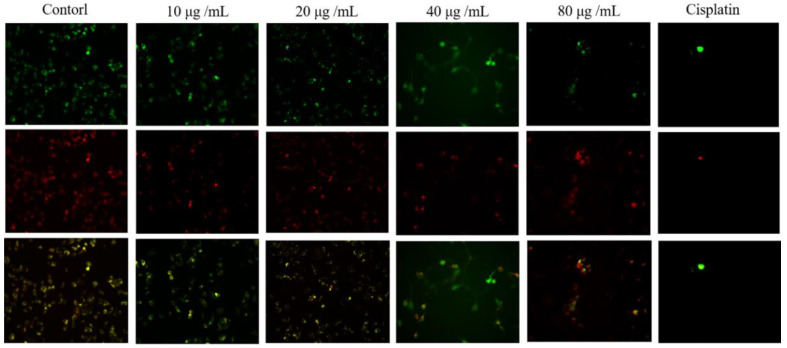
Effect of HSF on MMP levels in MCF-7 cells. Intracellular MMP levels were measured using Rh123 staining with an inverted fluorescence microscope. The phase contrast inverted microscopic images of the experimental groups (magnification, ×10).

The reduction in MMP levels indicates mitochondrial dysfunction. We found that adding different concentrations of HSF to MCF-7 cells decreased the intensity of green fluorescence gradually (Figure 4). Our results showed that the HSF could reduce the MMP level in MCF-7 cells and depolarize their mitochondrial membranes.

### 2.6. Effect of HSF on ROS Levels in MCF-7 Cells

**Figure 5:**
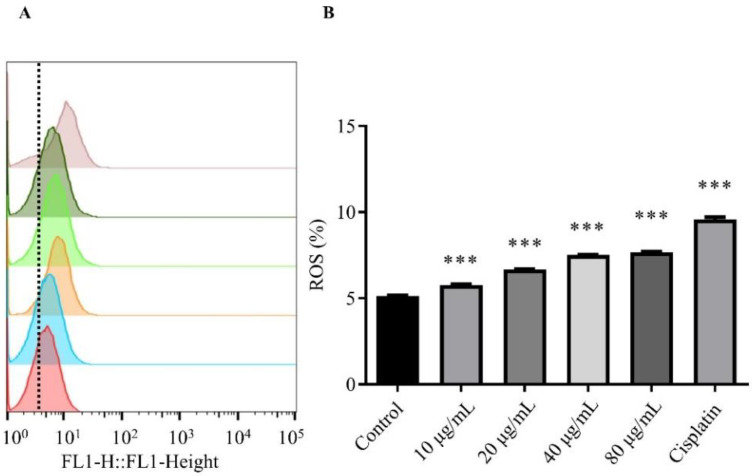
Effect of HSF on ROS levels in MCF-7 cells. ROS levels were measured using DCFH-DA staining via flow cytometry. The images from bottom to top indicate the control group, 10, 20, 40, and 80 μg/mL, and the positive drug group. (**A**) Flow cytometry for analysis of cellular ROS levels. (**B**) ROS%. *** *p* < 0.001 vs. control group.

Compared to the controls, DCFH-DA fluorescence during flow cytometry revealed a significant increase in ROS levels at all doses selected. The mean fluorescence intensity of DCFH-DA was estimated via flow cytometry to be 5.14, 5.77, 7.52, 7.67, and 9.58 in MCF-7 cells exposed to 0, 20, 40, and 80 μg/mL HSF and cisplatin, respectively (Figure 5). Compared to the control group, ROS accumulation was dose-dependent. The results showed that HSF could promote ROS accumulation in MCF-7 cells, and indicated that HSF induced oxidative stress and mitochondrial dysfunction during the apoptosis of MCF-7 cells.

### 2.7. The Expression of MCF-7 PI3K, AKT, p53, Caspase 9, and Cytochrome C 

**Figure 6:**
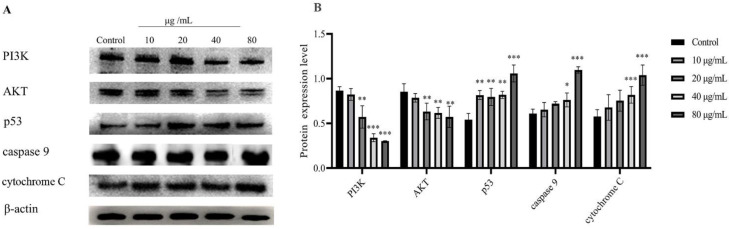
Apoptosis protein markers in MCF-7 cells treated with HSF of KR. (**A**) Immunoblots showing PI3K, AKT, p53, caspase 9, and cytochrome C expression are shown. (**B**) The densitometry of the bands, expressed in arbitrary densitometric units. The expression levels of PI3K, AKT, p53, caspase 9, and cytochrome C were determined by Western blotting analysis. β-actin was used as a loading control. * *p* < 0.05, ** *p* < 0.01, *** *p* < 0.001 vs. control group.

The process of apoptosis involves a complex cascade of proteolytic hydrolysis [45]. In comparison to the control group, an increase in the HSF concentration resulted in the significant downregulation of the expression of PI3K (80 μg/mL) and AKT (20, 40, and 80 μg/mL). The levels of important regulatory proteins, such as p53 (80 μg/mL), caspase 9 (80 μg/mL), and cytochrome C (40 μg/mL and 80 μg/mL), were significantly increased in the mitochondrial apoptotic pathway of MCF-7 cells from each treatment group, and their effects were dose-dependent (Figure 6).

## 3. Materials and Methods

### 3.1. Medicinal Plant Collection and Preparation 

Medicinal plants were obtained from Donghua Town, Chuxiong City, Chuxiong Prefecture, Yunnan Province, China (24°58′39′′ N, 101°29′16′′ E). The fresh plant was identified as KR by Professor Bin Qiu in October 2020 in Donghua Town, and the voucher specimen of KR (No. 20080416) was also deposited at the Key Laboratory of Southern Medicine Utilization, Yunnan University of Chinese Medicine (Kunming, China). The dried organic root of KR (1000 g) was crushed and immersed in 75% EtOH extraction for 1 h; the contents were extracted three times and filtered. Using a rotary evaporator and vacuum pump, the filtrate was mixed and dried to obtain the extract. The extracts (100 g) were suspended in H_2_O and then partitioned successively with petroleum ether (PET), ethyl acetate (EtoAc), and normal butanol (n-BuOH) to give PE (2.32 g), EtOAc (9.86 g), n-BuOH (7.76 g), and the final remainder, which was HSF (50.25 g). All extracted components were concentrated and lyophilized to obtain a powder. Cells were treated by dissolving extracts in dimethyl sulfoxide (DMSO) and sterilizing them with a 0.22 μm sterilizing filter. To obtain stock solutions with an appropriate concentration (to avoid DMSO-associated cellular toxicity). DMSO was detected at concentrations less than 0.2% under treatment conditions; their cytotoxicity was not detected at these concentrations [46].

### 3.2. Reagents and Assay kits

We obtained PET (analytical purity), EtoAc (analytical purity), n-BuOH (analytical purity), methanol (mass spectrometry grade), and acetonitrile (mass spectrometry grade) from Merck KGaA (Darmstadt, Germany). We obtained analytical grade PET, EtoAc, and n-BuOH from Merck KGaA (Darmstadt, Germany). We purchased chromatographic grade formic acid and DMSO from Sigma (St Louis, MO, USA). Cell Counting Kit-8 was purchased from Dojindo (Kyushu Island, JAPAN). A 0.22 μm sterilizing filter was purchased from Biosharp (Beijing, China). Annexin V-FITC and propidium iodide were purchased from BD (Franklin Lakes, NJ, USA). Fetal bovine serum (FBS) was purchased from Biological Industries (Israel). Dulbecco’s modified Eagle’s medium (DMEM), Roswell Park Memorial Institute (RPMI) 1640, and phosphate balanced solution (PBS) were purchased from Gibco, Thermo Fisher Scientific, Inc. (New York, NY, USA). β-actin (goat anti-rabbit) and RIPA lysis buffer were purchased from Solarbio (Beijing, China). PI3K, AKT, p53, cytochrome C, and caspase 9 antibodies were purchased from Bioss. Hoechst 33258, Mitochondrial Membrane Potential Assay Kit with Rhodamine 123 (Rh123), Reactive Oxygen Species Assay Kit (DCFH-DA), and bicinchoninic acid (BCA) assay kit were purchased from Beyotime (Shanghai, China). Polyvinylidene fluoride (PVDF) membranes (0.2 μm) were bought from Millipore (Burlington, MA, USA). The ECL Prime Western Blotting System was purchased from GE Healthcare.

### 3.3. Cell Culture 

DMEM supplemented with 10% FBS and 1% penicillin/streptomycin was used to routinely culture MCF-7, HeLa, and HepG2 cells. L02 and A549 cells were routinely cultured in RPMI 1640 medium supplemented with 10% FBS and 1% penicillin/streptomycin. Cells were incubated at 37 °C with 5% CO_2_ in a humidified atmosphere. Logarithmically growing cells were used in all experiments. All the human cell lines were obtained from the American Type Culture Collection (ATCC, Rockville, MD, USA).

### 3.4. Cell Viability Assay 

After the dosing process, the levels of cell viability and proliferation were determined using the CCK-8 kit. Each cell line (1 × 10^4^ cells/well) was incubated in 96-well plates. The medium was removed after 24 h and the cells were washed with sterile PBS. At 37 °C, cells were treated with 0, 10, 20, 60, and 80 μg/mL of different medicinal KR ingredients (PET, EtoAc, n-BuOH, and H_2_O) for 24 h. As positive and negative controls, cisplatin and DMSO (at concentrations less than 0.2%) were used. Incubation was conducted for 2 h after removing mixtures, and cells were washed twice with 100 μL of sterile PBS. Next, each well was filled with 100 µL of culture medium containing 10% CCK-8 and incubated for 2 h at 37 °C. To measure 450 nm absorbance, we used SpectraMax M Series Microplate Readers (Molecular Devices, Silicon Valley, CA, USA). 

Based on the results obtained using the CCK-8 kit, cells exhibiting optimal inhibitory effects and responses to drug components were selected for subsequent experiments. MCF-7 cells and HSF were selected for use in the next experiment.

### 3.5. Liquid Chromatography–Mass Spectrometry (LC–MS) Analysis

HSF were detected using LC-MS on a 6540 UHD accurate-mass quadrupole–time of flight (Q-TOP) spectrometer equipped with a Dual AJS electrospray ionization (ESI, Agilent Technologies, Inc., Santa Clara, CA, USA). The UHPLC conditions were as follows. Column: Ultimate Phenyl-Ether (250 mm × 4.6 mm, 5 μm) at 30 °C; injection volumes were 10 μL and flow rate was 1.0 mL/min. The mobile phase consisted of eluent A (acetonitrile) and eluent B (0.1% formic acid, *v*/*v*). The elution gradient for A was 0–0.1 min, 10% A; 0.1–5 min, 10–25% A; 5–15 min, 25–35% A; 15–25 min, 35–50% A; 25–35 min, 50–65% A; 35–45 min, 65–85% A; 45–55 min, 85–100% A; 55–65 min, 100% A. The mass spectrometry analysis was performed using a triple-quadrupole detector (TQD) mass spectrometer (Agilent) equipped with an electrospray ionization source (ESI+: positive ion mode for compounds. ESI-: negative ion mode for compounds). Setting: 100 °C source temperature, 3.5 kV capillary voltage, 400 °C desolvation gas temperature; cone voltage, 20 V; and flow rate, 700 L/h. A range of 150–1000 *m*/*z* was recorded for the MS spectra.

### 3.6. Morphological Apoptosis

An effective way to observe the morphology of apoptotic cells is to stain them with Hoechst 33,258. The effects of HSF were investigated with Hoechst 33,258 staining to determine whether cell death was induced by HSF. In 6-well plates, 2 × 10^5^ MCF-7 cells were seeded per well. The medium was removed after 24 h and the cells were washed with sterile PBS. At 37 °C, cells were treated with 0, 10, 20, 60, and 80 μg/mL HSF for 24 h. As positive and negative controls, cisplatin and DMSO (at concentrations less than 0.2%) were used. Next, cell morphology was analyzed using Hoechst 33,342 solution. Using a fluorescence microscope (Zeiss, Germany), morphological changes in the nucleus were examined. The nuclei of normal cells were blue, while apoptotic nuclei were in the dense and hyperchromatic state, or a fragment-like dense and hyperchromatic state, and had a whitish color.

### 3.7. Apoptosis Analysis

An Annexin-V-FITC Apoptosis Detection Kit was used to measure the extent of apoptotic cell death induced by HSF treatment. In 6-well plates, 2 × 10^5^ MCF-7 cells were seeded per well. The medium was removed after 24 h and the cells were washed with sterile PBS. At 37 °C, cells were treated with 0, 10, 20, 60, and 80 μg/mL HSF for 24 h. As positive and negative controls, cisplatin and DMSO (at concentrations less than 0.2%) were used. Next, in centrifugation tubes, the adherent and incubated cells were collected along with the digested cells digested by 0.25% EDTA-free trypsin, and binding buffer was added after three washes with chilled PBS. Next, a BD FACSAriaTM III Cell Sorter flow cytometer (BD Biosciences, Franklin Lakes, NJ, USA) was used to examine the cells incubated with FITC-labeled Annexin-V and PI for 20 min in the dark. Analysis was performed using FlowJo V10 software.

### 3.8. Measurement of Intracellular MMP

MMP is one of the important indicators of the maintenance of normal mitochondrial morphology and function [47]. A decrease in the MMP level was proven to be a significant indicator of early cell apoptosis [48]. Therefore, we detected the changes in MMP levels by determining the fluorescence intensity ratio between green and red and, when HSF was applied to cells, these levels decreased notably when compared with those in the control group. The alteration in the MMP level was examined by using the Rh123 Assay Kit. In 6-well plates, 2 × 10^5^ MCF-7 cells were seeded per well. The medium was removed after 24 h and the cells were washed with sterile PBS. At 37 °C, cells were treated with 0, 10, 20, 60, and 80 μg/mL HSF for 24 h. As positive and negative controls, cisplatin and DMSO (at concentrations less than 0.2%) were used. The next day, cells were treated with Rh123 staining solution at 37 °C in a 5% CO_2_ incubator for 30 min after the supernatants were removed from the culture dishes. Thereafter, cells were washed with PBS three times, and the cells were photographed via fluorescence microscopy (Zeiss, Aalen, Germany).

### 3.9. Measurement of Intracellular ROS

The occurrence, development, and treatment of tumors are closely related to ROS, which plays an important role in cell signaling [49,50]. ROS were detected with DCFH-DA probe. In 6-well plates, 2 × 10^5^ MCF-7 cells were seeded per well. The medium was removed after 24 h and the cells were washed with sterile PBS. At 37 °C, cells were treated with 0, 10, 20, 60, and 80 μg/mL HSF for 24 h. As positive and negative controls, cisplatin and DMSO (at concentrations less than 0.2%) were used. Then, we suspended cells in DCFH-DA and incubated them for 20 min at 37 °C. A flow cytometer, the BD FACSAria^TM^ III Cell Sorter, was used to quantify ROS by measuring the intracellular fluorescent intensity of the cells resuspended in PBS (BD Biosciences). The data were then analyzed with FlowJo V10.

### 3.10. Western Blot Analysis

Incubation of synchronized MCF-7 cells with different concentrations of HSF for 24 h was followed by lysis with RIPA buffer containing protease inhibitors and phosphatase inhibitors. Assaying the protein concentration was carried out using a BCA protein assay kit after centrifugation at 4 °C and 12,000× *g* for 10 min. The cell extracts were added to the sample loading buffer and denatured at 99 °C for 10 min. Mixing proteins with buffer, electrophoresing them on 10% SDS-PAGE gels, and transferring them onto PVDF membranes were the steps used. At 4 °C overnight, the membranes were probed with primary antibodies (1:1000) after blocking with 5% milk in TBST for 2 h at room temperature. A diluted secondary antibody (1:8000) was incubated with the membranes for 1 h at room temperature each and then washed 3 times for 5 min with TBST. Target proteins were detected using a chemiluminescent gel imaging system (Tanon, Shanghai, China) and blots were visualized using ImageJ software (National Institutes of Health, Bethesda, MD, USA).

### 3.11. Statistical Analysis 

All cell culture assays were performed using triplicate samples and repeated at least three times. GraphPad Prism 9.0 software (GraphPad Software) was used for data analysis. Data were represented as means ± standard deviation (SD) values unless indicated otherwise. Differences between groups were evaluated using the Student’s *t*-test or one-way analysis of variance (ANOVA). *p* < 0.05 was set as the threshold value for statistical significance.

## 4. Discussion

Several of the chemical components of KR have been shown to have anti-tumor properties. This study examined how KR effects tumor cells. The extracted fractions had cytotoxic effects against A549, HepG2, MCF-7, and HeLa tumor cells. However, when KR was applied to L02 cells, no toxicity was observed. These results show that its inhibitory effect is selective and that HSF of KR has the best inhibitory effect against breast cancer cells. Apoptosis induction and inhibition of proliferation were examined as well as potential molecular mechanisms of action. It was demonstrated that HSF of KR significantly decreased the activity of MCF-7 cells. 

Subsequently, UHPLC-MS was utilized to identify the chemical components of HSF against breast cancer. Interestingly, all the eight identified compounds have been reported as having anti-tumor effects, while calycosin, aloe emodin, rhein, and maackiain have been reported as having anti-breast cancer effects. Phytoestrogens such as calycosin are thought to exhibit anti-cancer effects through different molecular pathways. They affect estrogen receptors, cellular signaling pathways, cell cycle, apoptosis, and steroid synthesis, and induce DNA damage in cancer cells [51]. Maackiain could suppress breast cancer cell proliferation, lesion formation, migration, and invasion [31]. Breast cancer cells were inhibited from invading and migrating by aloe emodin, which inhibited pro-tumorigenic cytokine expression [30]. In combination with rhein and atezolizumab, spleen and tumor CD8^+^ T cell ratios were significantly increased, and the levels of TNF-α, interleukin-6, and various apoptosis factors in the serum under combined therapy were significantly higher than those in other treatment groups, and the growth of breast tumors was effectively inhibited. Aezolizumab’s effects are enhanced by rhein in breast cancer treatments [52]. There is a possible correlation between the levels of these eight compounds and the cytotoxic effects of HSF on MCF-7 cells as well as its ability to induce apoptosis.

Then, assays with Annexin V-FITC/PI and Hoechst 33258 staining were conducted to discover whether HSF caused apoptosis. An increase in apoptosis rates and significant changes in cell morphology were found. We have further investigated the mechanism of apoptosis. The decrease in MMP levels is one of the characteristics of early apoptosis in tumor cells. The depolarization of the mitochondrial membrane leads to the release of pro-apoptotic factors and the activation of apoptotic protease in mitochondria, which finally induces apoptosis. Meanwhile, studies have reported that mitochondria are the main source of ROS in cells. The ROS level is closely related to the occurrence of apoptosis. The excessive production of ROS causes oxidative damage to important cellular molecules and structures (such as lipids, proteins, and DNA), resulting in mitochondrial dysfunction, endogenous apoptosis pathway activation, and ultimately, apoptosis [53,54]. Moreover, a variety of anti-cancer drugs caused tumor cells to produce ROS, which in turn triggered mitochondrion-mediated apoptosis [55]. The drug doxorubicin also induces mitochondrial dysfunction and apoptosis [56,57]. Our analysis by fluorescence microscopy showed that the HSF of KR could downregulate the mitochondrial membrane potential of MCF-7 cells and the flow cytometry analysis showed that the intracellular ROS content was increased, suggesting that the HSF of KR could induce the apoptosis of MCF-7 cells.

In addition to the above relevant changes, molecular markers are altered during apoptosis. The key role played by the PI3K/AKT pathway in cancer development is well known [58], such as in cell proliferation, growth, invasion, migration, and angiogenesis [59]. PI3K is a member of the phospholipid kinase family. It has gained considerable attention in the field of cancer research. In addition to regulating cell survival, proliferation, migration, apoptosis, and angiogenesis, AKT plays a key role in numerous cellular processes [60,61]. First, the AKT pathway is considered to be an important signal pathway, which regulates cell proliferation and apoptosis by regulating the p53 tumor suppressor gene and caspase 3 signal pathway. Next, a variety of cellular stresses can activate the tumor suppressor protein p53, including cell death, DNA damage, oxidative stress, and hypoxia [62,63]. Tan et al. verified that *Polyporus umbellatus* extract can enhance the expression of caspase 3, regulate anti-apoptotic and pro-apoptotic signals of AKT, and induce apoptosis in breast cancer cells, and it is a significant inhibitor of growth, invasion, and metastasis [64]. As a proto-oncogene, PI3K will be phosphorylated and the downstream effector AKT will be activated when it is affected by a variety of adverse factors such as cytokines, physical radiation, and chemical stimulation. The activated AKT will activate the downstream targeted molecules, leading to the proliferation and migration of cancer cells [65]. Our results show that the HSF of KR could effectively downregulate the expression of PI3K and AKT proteins, and upregulate the expression of p53. 

Moreover, caspase 9 is activated in cells; this initiates an inclusive cascade and promotes the transfer of cytochrome C from the mitochondrial inner membrane to the cytoplasm [66,67]. A previous study has shown that the decrease terpenoid indole alkaloids in mitochondrial membrane potential caused by mitochondrial dysfunction leads to the opening of the mitochondrial permeability transition pore (mPTP), which releases cytochrome C from the mitochondria into the cytoplasm [68]. Using Western blotting, we found that HSF of KR upregulated caspase 9 and cytochrome C expression, indicating that HSF induces mitochondrion-mediated apoptosis in breast cancer cells.

**Figure 7:**
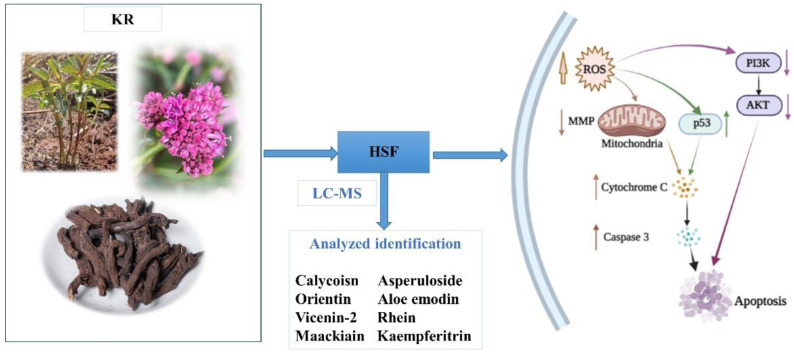
Pathway of apoptosis induced by HSF in MCF-7 cells.

Upon aberrant ROS production, pro-apoptotic and anti-apoptotic proteins are altered, MMP is lost, cytochrome C is released, the mitochondrial membrane is damaged, and, eventually, cell death through the mitochondrial apoptotic pathway occurs [69]. Reactive oxygen species released by p53 affect mitochondrial membrane potential and trigger cytochrome C-independent apoptosis, which is inhibited by the presence of Bcl-2. In addition, the elevation in ROS levels activates PI3K/AKT signaling [70]. As shown in Figure 7, the hypothetical mechanism required for HSF treatment can be illustrated schematically. It appears that the mitochondrial pathway might be associated with HSF-regulated proliferation and apoptosis in MCF-7 cells. 

In conclusion, our data analysis demonstrated that HSF can inhibit proliferation and actuate apoptosis in MCF-7 cells. The outcomes additionally exhibited that HSF initiated MCF-7 cell apoptosis via the mitochondrion-mediated pathway. These findings may provide insights to better understand the potential mechanisms of the anti-cancer effects of HSF and assist with finding elective therapy procedures for breast cancer. Animal models and patient studies were absent from the present study, which is its primary limitation. Consequently, further investigation of these extracts’ active compounds is necessary, and to develop these herbs as therapeutic agents for human breast cancer, in vivo experiments are required.

## Figures and Tables

**Table 1:** An analysis of the IC_50_ values in different cells treated with PET, EtoAc, n-BuOH, and HSF.

Cell Lines	IC50 (μg/mL)			
	PET	EtoAc	n-BuOH	HSF
A549	191.85 ± 3.18	186.86 ± 4.19	60.63 ± 0.35	1155.107 ± 3.41
HepG2	186.50 ± 3.03	209.81 ± 2.66	271.71 ± 2.13	183.82 ± 1.37
MCF-7	75.295 ± 2.66	60.93 ± 3.46	104.29 ± 2.55	28.84 ± 0.60
HeLa	64.92 ± 1.57	82.64 ± 2.54	71.21 ± 2.37	66.90 ± 1.97

**Table 2:** Phytochemical analyses and identified compound profiles in HSF of KR.

Name	References	Mode	Observed RT (min)	Neutral Mass	Observed *m*/*z*	Mass Error (ppm)	MS/MS	Formula	Relative Contents (%)
Calycosin	[38]	(M + H)	10.5467	284.0685	284.0684	0.13	269.0666, 213.2516	C_16_H_12_O_5_	4.98
Asperuloside	[39]	(M − H)	4.9759	414.1162	414.1169	1.15	146.9253, 190.9838	C_18_H_22_O_11_	1.30
Orientin	[40]	(M − H)	6.3904	448.1001	448.1006	1.33	327.0541, 357.0070	C_21_H_20_O_11_	1.87
Aloe emodin	[41]	(M − H)	22.3318	270.0528	270.0523	1.9	225.0148, 241.0063	C_15_H_10_O_5_	3.56
Vicenin-2	[42]	(M + H)	5.7913	594.1584	595.1651	0.11	325.1827, 337.1037	C_27_H_30_O_15_	1.03
Rhein	[41]	(M − H)	56.6358	284.0321	284.0321	0.81	239.0362, 257.0376	C_15_H_8_O_6_	18.09
Maackiain	[43]	(M − H)	27.8311	284.0685	284.0685	0.49	224.0256, 175.7748, 163.0731	C_16_H_12_O_5_	60.81
Kaempferitrin	[44]	(M − H)	13.2285	578.1636	578.1636	0.42	255.1002, 430.0110, 285.0406	C_27_O_30_H_14_	8.36

Note: The purity of the above eight compounds referenced the standard compounds in addition to 94.28% of vicenin-2, the other seven are ≥98%. For the references, the methods used to determine the compounds were all standard controls. Compounds in the table are tentative without comparison with reference samples.

## Data Availability

All the data used to support the results of this research can be obtained from the corresponding author.

## Sample Availability

Samples of the compounds are not available from the authors.

## Abbreviations

butanol	*n*-BuOH
Cell Counting Kit-8	CCK-8
ethyl acetate	EtoAc
H_2_O-soluble fraction	HSF
*Knoxia roxburghii* (Spreng.) M. A. Rau	KR
mitochondrial transmembrane potential	MMP
mitochondrial permeability transition pore	mPTP
petroleum ether	PET
reactive oxygen species	ROS
terpenoid indole alkaloids	TIAs
